# Supplementary material for: Molecular insights into intra-complex signal transmission during stressosome activation
Source: Commun Biol. 2022 Jun 27;5:621. doi: 10.1038/s42003-022-03549-9 (PMC9237128; doi:10.1038/s42003-022-03549-9)
Supplement: Supplementary file 2 — Supplemental materials [file 42003_2022_3549_MOESM2_ESM.pdf]

SUPPLEMENTARY INFORMATION

Molecular insights into intra-complex signal transmission during stressosome activation

Algirdas Miksys[1]*, Lifei Fu[1]*, M. Gregor Madej[1]*, Duarte Guerreiro[2], Susann Kaltwasser[3], Maria Conway[4], Sema Ejder[5], Astrid Bruckmann[6], Jon Marles-Wright[5], Richard J. Lewis[7, §], Conor O'Byrne[2], Jan Pané-Farré[4, #], and Christine Ziegler[1, #]

1) Department of Biophysics II / Structural Biology, University of Regensburg, Regensburg 93053, Germany
2) Bacterial Stress Response Group, Microbiology, School of Natural Sciences & Ryan Institute, National University of Ireland Galway, Galway, Ireland, H91 TK33.
3) Department of Structural Biology, Max-Planck Institute of Biophysics, Frankfurt am Main 60438, Germany
4) Center for Synthetic Microbiology (SYNMIKRO) and Department of Chemistry, Philipps-University Marburg, 35043 Marburg, Germany
5) School of Natural and Environmental Sciences, Newcastle University, Newcastle upon Tyne, NE1 7RU, UK
6) Department of Biochemistry I, University of Regensburg, Regensburg 93053, Germany
7) Newcastle University Biosciences Institute, Faculty of Medical Sciences, Newcastle University, Newcastle upon Tyne NE2 4HH, UK

§) Current address: The Royal Society for the Protection of Birds, The Lodge, Potton Road, Sandy, Bedfordshire SG19 2DL

# Correspondence:

email: christine.ziegler@biologie.uni-regensburg.de telephone: +49-941 943 3030

email: jan.panefarre@chemie.uni-marburg.de, telephone: +49-6421 28 22211

SUPPLEMENTARY FIGURES

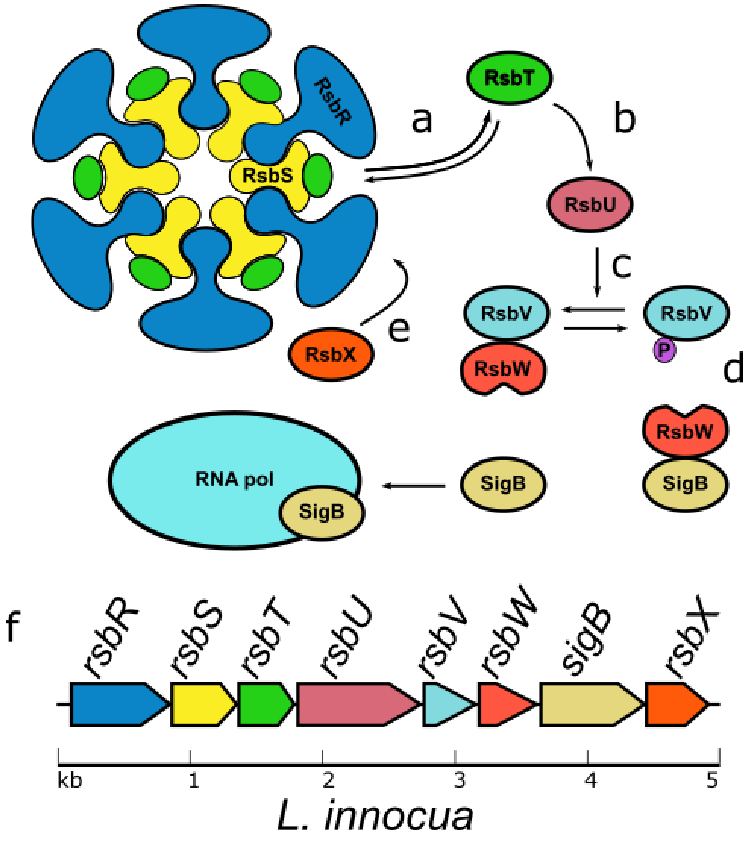

Supplementary figure 1. **Organization of the *Listeria innocua* stressosome and activation model described in *B. subtilis*.** Upon sensing stress, the stressosome core proteins, RsbR and RsbS, are phosphorylated by RsbT (a). RsbT dissociates from the core of the stressosome complex and stimulates RsbU (b), which in turns dephosphorylates RsbV (c). The dephosphorylated RsbV complex sequesters RsbW from the SigB-RsbW complex (d), leaving SigB free to associate with the RNA polymerase. The stressosome complex is reset by the phosphatase RsbX (e). In gram-positive bacteria, the sigB associated operon is conserved

between different genera, the general arrangement as present in *B. subtilis* and *Listeria* species.

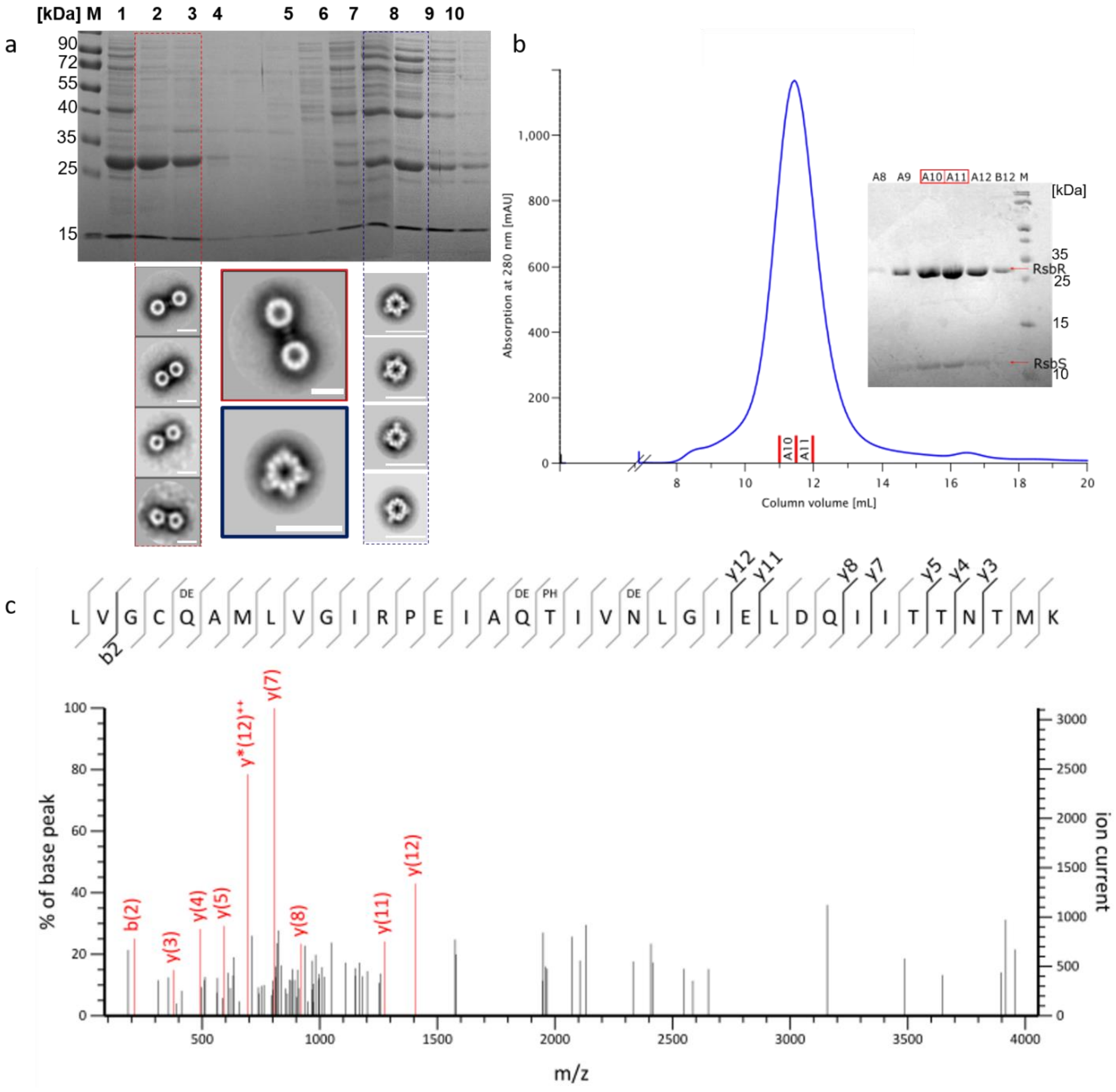

Supplementary figure 2. **Purification of the *Li*RsbRS complex.** (a) SDS-PAGE (top) of the anion exchange purification of *Li*RsbS using a DEAE column. Complexes isolated from the first fraction (red outline) and second fraction (blue outline) of the anion exchange purification have been analyzed by negative stain single particle EM (bottom). 2D classification shows the 1st fraction forming dimeric aggregates, whereas the 2nd fraction tends to remain monomeric. All scalebars are 200 Å. The full size gels can be found in Supplementary figure 13. (b) The final size exclusion chromatography profile of the 2nd fraction of the *Li*RsbRS purification used for cryo-EM structure determination, resulting in a monodisperse peak. The indicated

fractions have been analyzed by SDS-PAGE (inset) to confirm the purity of the complex. (c) The fragmentation spectrum of a peptide of RsbR containing a phosphate modification. Analysis of RsbR by ESI-MS-MS revealed a peptide of 3975.0648 Da (Δm/z=1.75 ppm), indicating the presence of phosphorylation. The parent peptide is shown at the right-most side of the spectrum, and its fragmentation products indicate that Thr241 (red arrow) is phosphorylated (PH) The fragment in yellow shows a neutral loss of a phosphate group on the peptide.

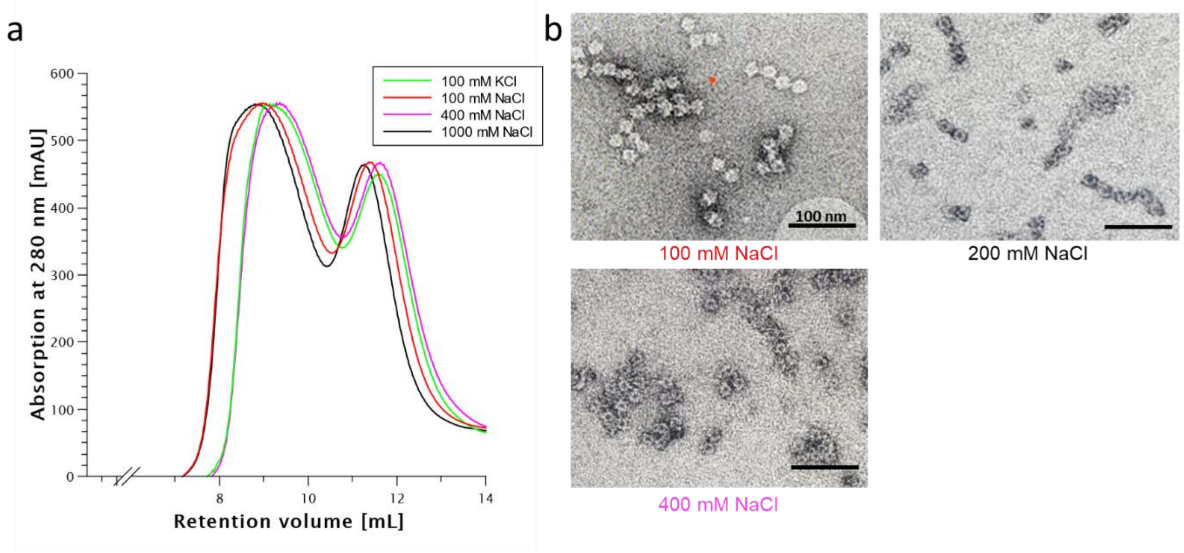

Supplementary figure 3. **Effects of salt concentration on the stability of the *Li*RS complex.** (a) SEC profiles of *Li*RsbRS purified in the presence of different amounts of NaCl or KCl. An anion exchange purified *Li*RsbRS complex from the early eluting fraction was injected onto a Superose 6 Increase (GE Healthcare) column equilibrated with buffers containing one of the salt concentrations listed in the legend of (a). While a fraction of the complex elutes in the void volume of the column indicating aggregation, the complex retains its stability over a wide range of salt concentrations. (b) Negative stain EM images of *Li*RsbRS complexes incubated with different salt concentrations. As salt concentration increases, the complex tends to aggregate more on the negative stain grids. The scale bar in all images is 100 nm.

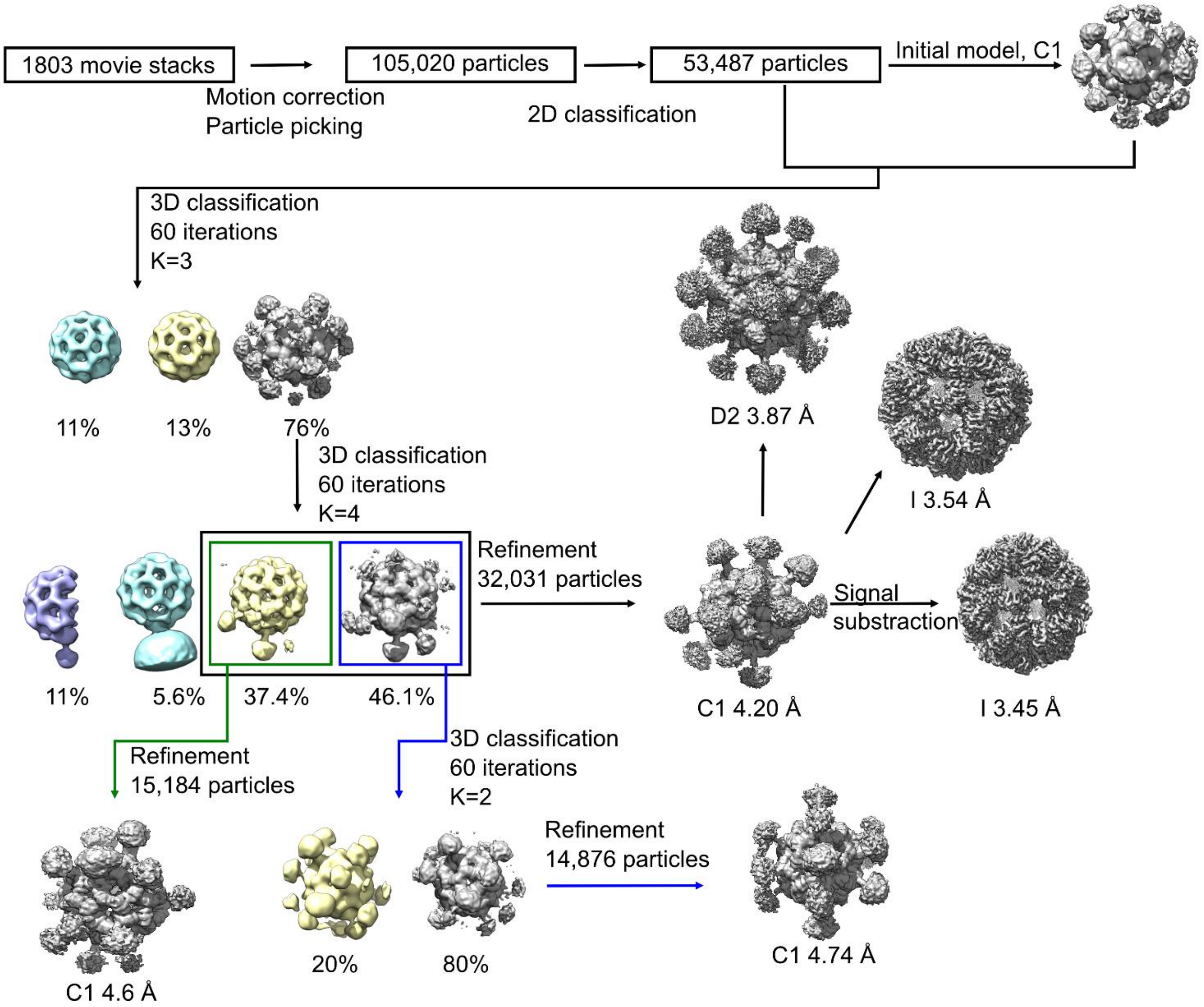

Supplementary figure 4. **RELION data processing workflow for the *Li*RsbRS stressosome complex.** The dataset was cleaned up using 2D classification to identify and remove the broken particles. The remaining particles were then subjected to 3D classification using an initial model generated from the data. Two 3D classes containing 37.4 % (green rectangle) and 46.1 % (blue rectangle) of the particles were investigated further. The class containing 37.4% of the particles was refined to a unsymmetrized model at a resolution of 4.6 Å. The other 3D class was further classified to establish the positions of the RsbR turrets in the complex (bottom). The particles in both largest classes were pooled for further analysis and refinement. Afterwards, different symmetry operators were used to improve features of the

core and turret regions of the complex. Subtracting the signal of the turrets was also done to further improve the resolution in the core region.

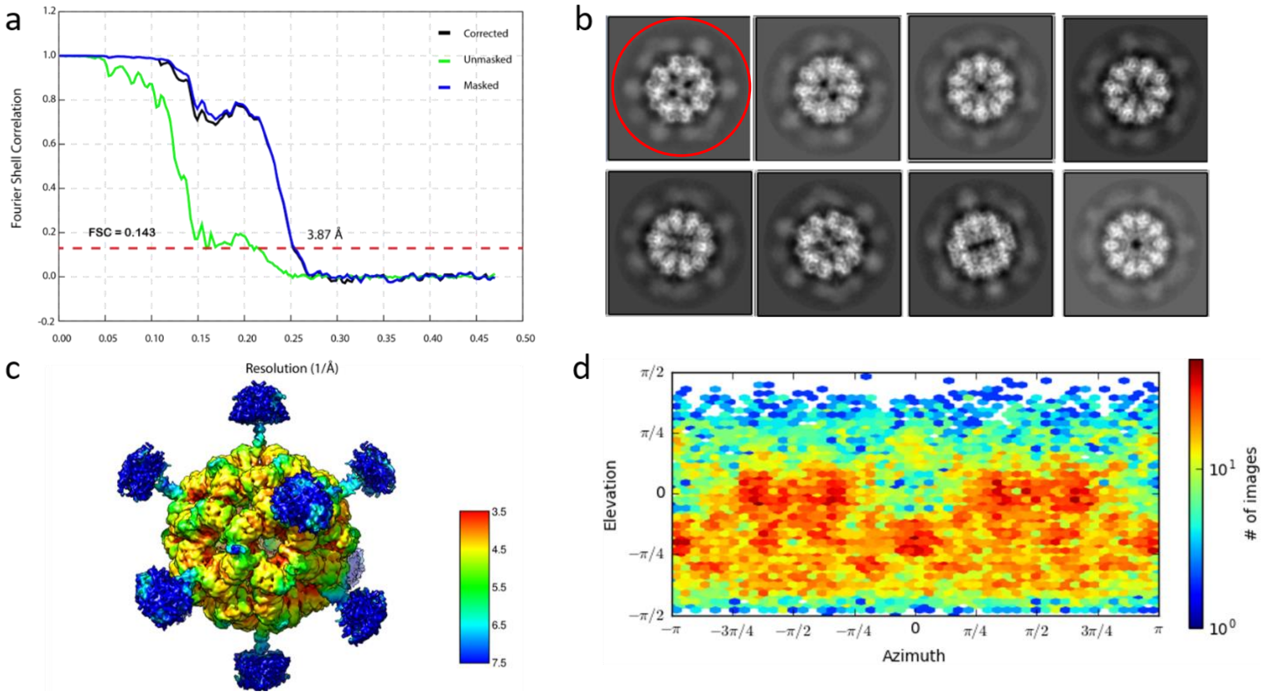

Supplementary figure 5**. The results of processing the *Li*RsbRS dataset in D2.** (a) The FSC plot showing the resolution estimate of the *Li*RsbRS complex with various masking procedures implemented. (b) Representative 2D class averages from the cleaned dataset. Secondary structure features of the core are more prominent, whereas the turret intensities are weaker. The circular mask used for 2D classification is 300 Å in diameter (red circle). (c) Local resolution map displaying the higher resolution of the core and the less resolved turrets. The heatmap shows the local resolution of the map in Angstrom (Å). (d) The angular distribution plot for the particles used in reconstruction.

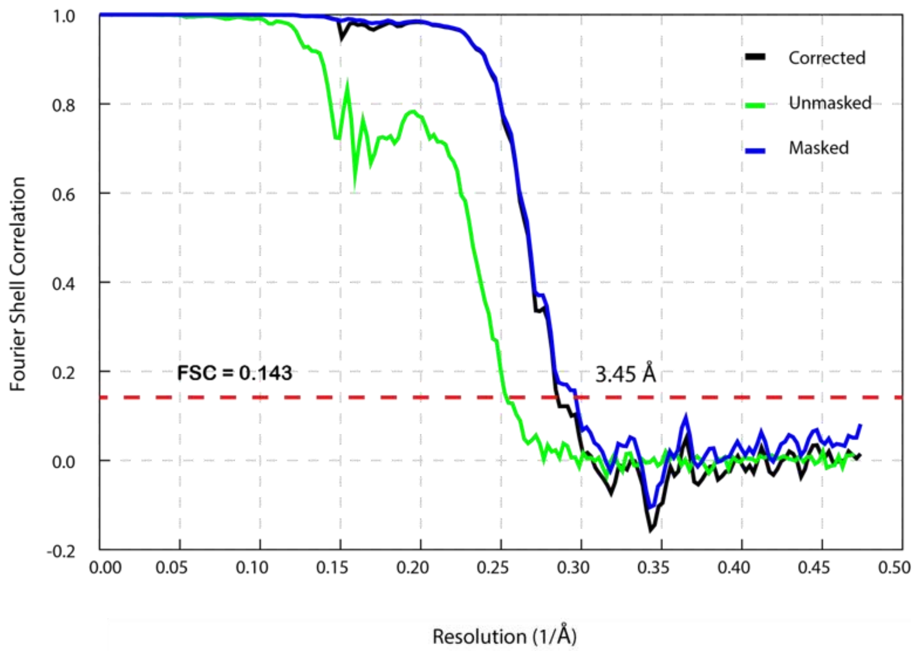

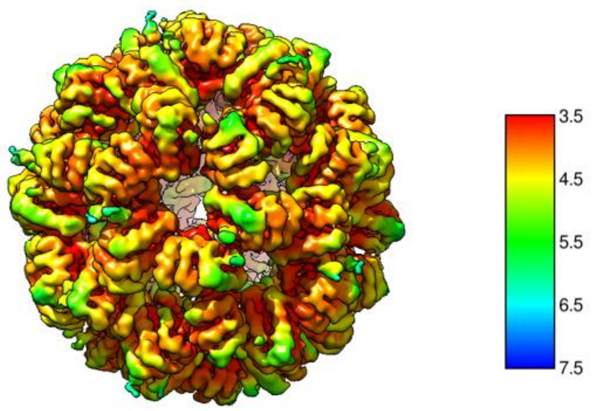

Supplementary figure 6. **Resolution of the STAS core of *Li*RsbRS.** An icosahedral reconstruction of *Li*RsbRS with the turret density subtracted was performed. The FSC plot (top) obtained from RELION indicates that the resolution improvement was in the core region was significant. The map generated by RELION local-res (bottom) indicates that there is variability in the overall resolution of the core, with the outward parts appearing less well resolved, likely reflecting their inherent flexibility. The heatmap shows the local resolution of the map in Angstrom (Å).

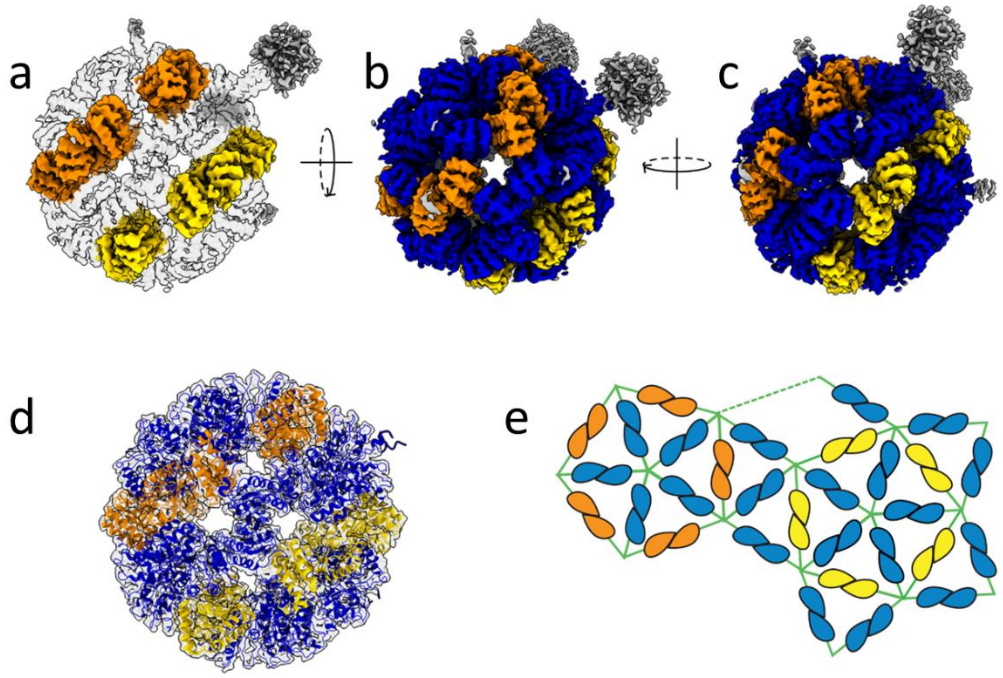

Supplementary figure 7. **Comparison of the *L. monocytogenes* *Lm*RsbRS complex assembly to the one proposed in Figure 1. a-d**: Asymmetric cryo-EM density map of the *Lm*RsbRS stressosome complex (EMD-4508) at a resolution of 4.21 Å, colored according to the deposited model (PDB: 6QCM). The STAS domains of RsbS are colored in orange and yellow, the RsbR STAS domains are displayed as a transparent or blue surface. (a) Semi-transparent depiction, highlighting the ring-shaped arrangement of RsbS in the complex. (b) Tilted view of (a), centered on a five-fold vertex of the complex. (c) Tilted view of (b), centered on a three-fold vertex. (d) The molecular model of the STAS domain core of the complex, fitted into the cryo-EM density map. For simplicity, the RsbR turret densities are not shown. (e) The unfolded view of the *L. monocytogenes* stressosome complex, depicting the arrangement of RsbS and RsbR in a conventionalized way. The two rings of RsbS dimers are shown in orange and yellow, RsbR dimers are depicted in blue. Both orange and yellow RsbS dimers surround a five-fold vertex respectively, which is exclusively built from RsbR dimers. This arrangement

of subunits in the complex differs from the arrangements of the *Vibrio vulnificus*, *Listeria innocua* and *Bacillus subtilis* complexes.

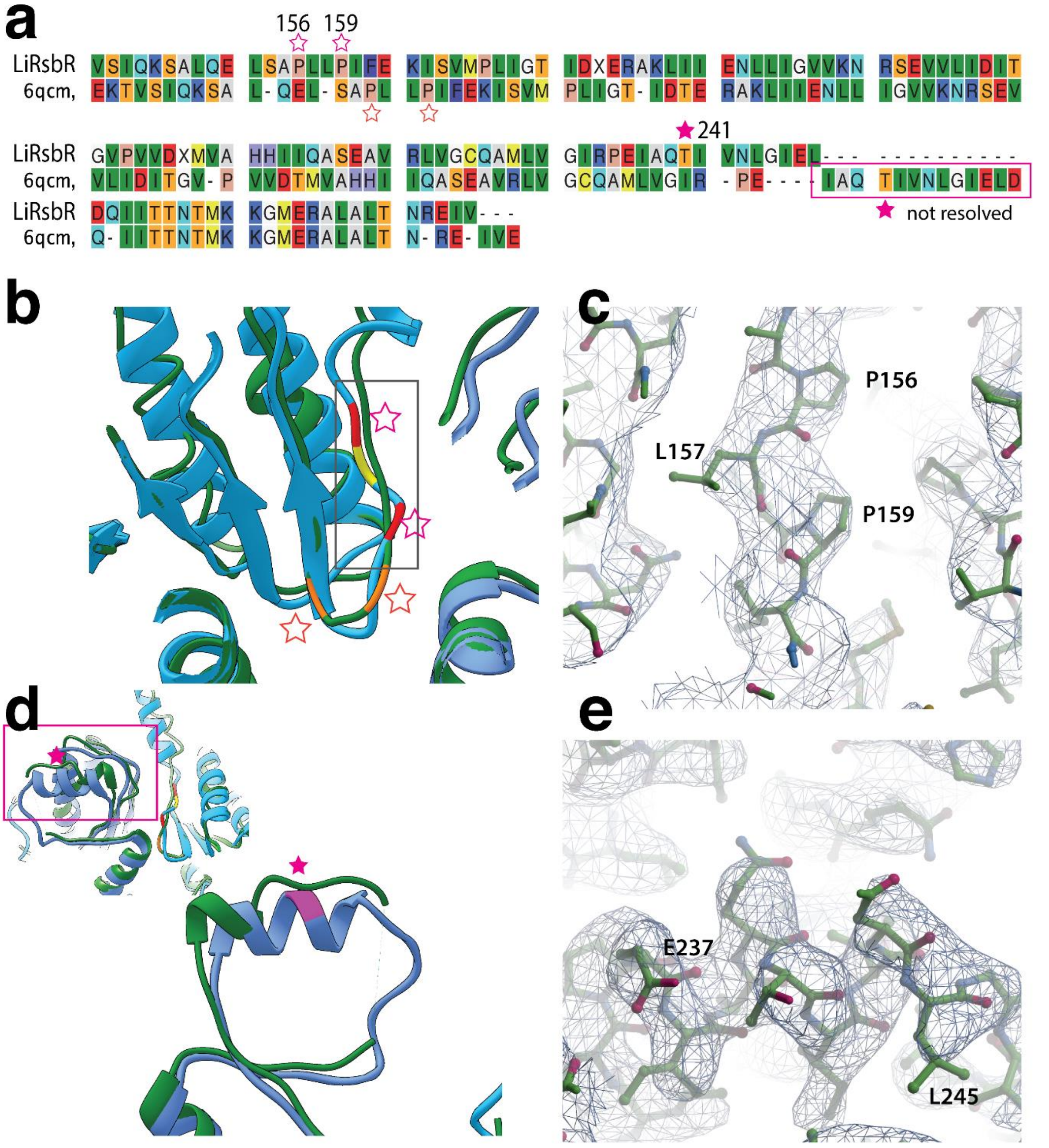

Supplementary figure 8. **Structural differences between the *L. innocua* and *L. monocytogenes* RsbR.** (a) Alignment of the structural features present in *Li*RsbR (PDB 7B0U) and *Lm*RsbR (PDB ID: 6QCM). (b) the placement of proline residues in *Lm*RsbR (green) and *Li*RsbR (blue). The magenta and orange stars indicate the relative positions of Pro156 and Pro159 in the structures and in (a). (c) The *Li*RsbR cryo-EM density map in the corresponding region with Pro156 and Pro159. (d) The helical region containing Thr241 (magenta star) is

helical in *Li*RsbR (blue), further shown in the EM density map (e), whereas the region is a interpreted as a loop in *Lm*RsbR (green).

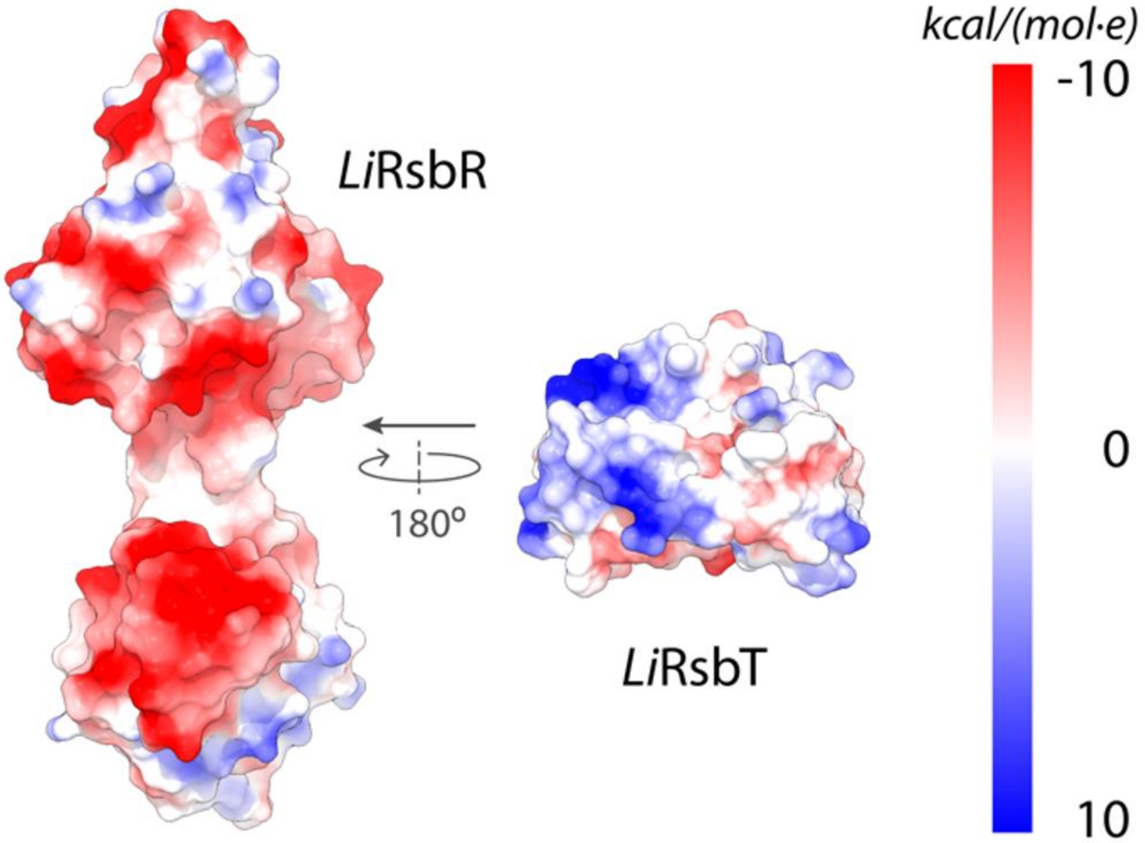

Supplementary figure 9. **Coulombic surface of *Li*RsbR:*Li*RsbT**. The charge distribution indicates a bidental binding for the *Li*RsbR:*Li*RsbT complex. The complementary charges on the potential interaction surface are very pronounced. The Coulombic surface was generated using the standard procedure in UCSF Chimera with default settings.

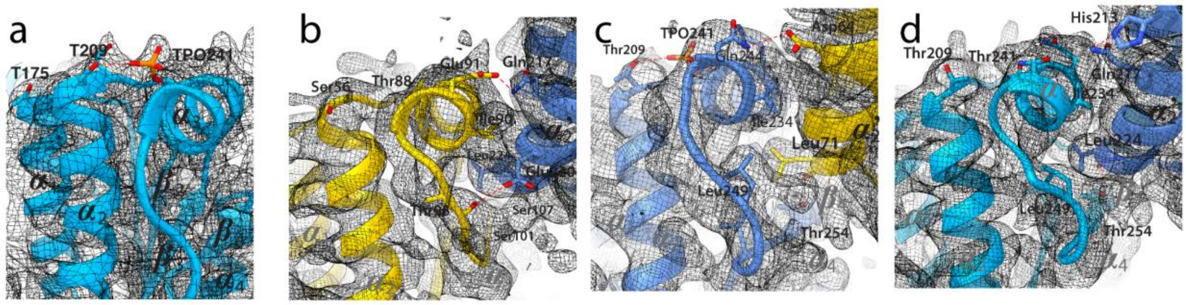

Supplementary figure 10. **The fit of the model and the data regarding the features described in the main text.** The density around the TPO241 (a) on RsbR STAS, (b) the features around the STAS-STAS interfaces between RsbR-RsbS, (c) RsbS-RsbR, (d) RsbR-RsbR interface of RsbR-RsbS.

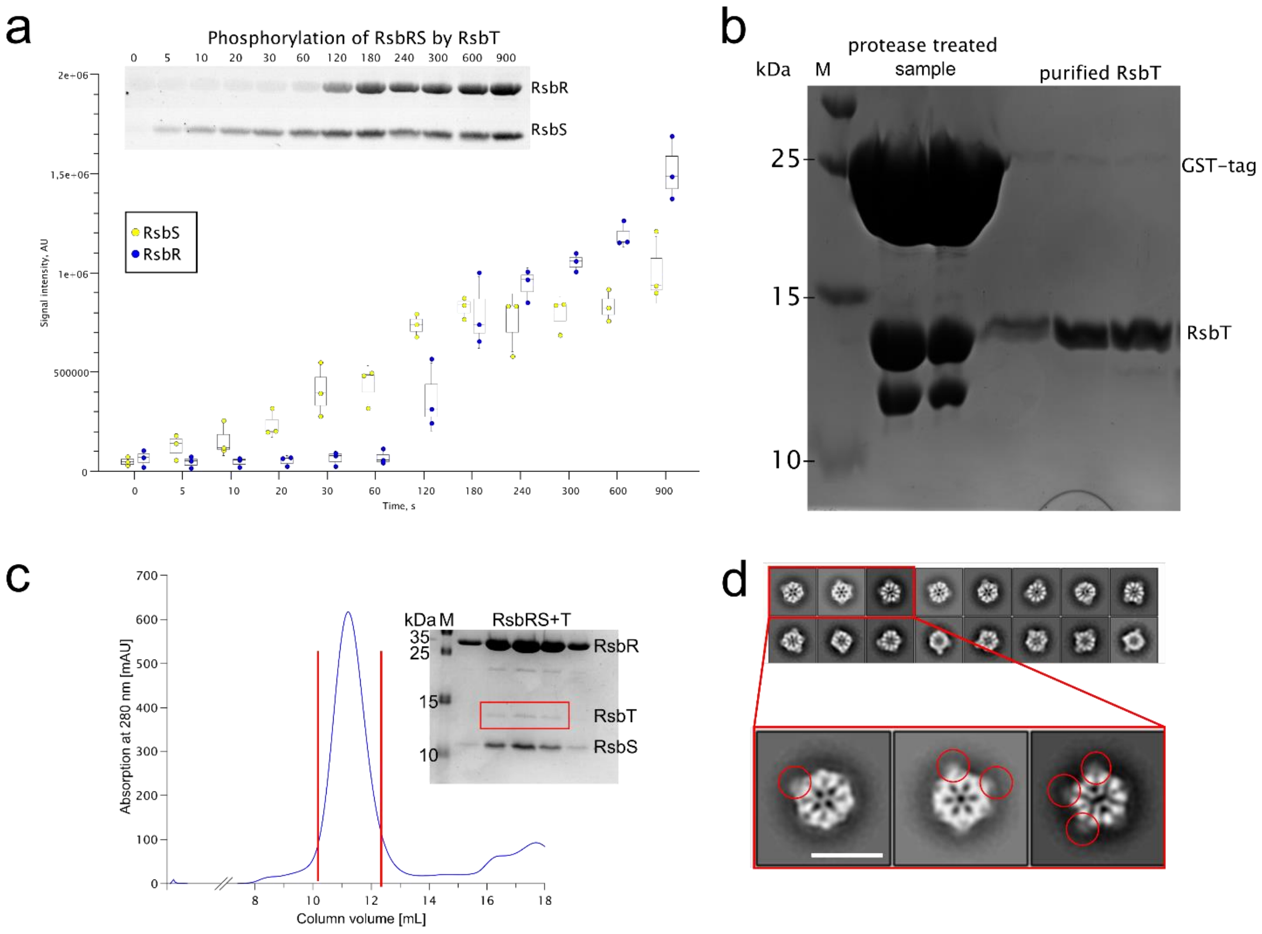

Supplementary figure 11. **In vitro phosphorylation of RsbRS by RsbT and ternary complex assembly.** (a) The phosphorylation levels at various timepoints of RsbRS complex phosphorylation by RsbT as determined by SDS-PAGE with phosphorylation specific ProQ diamond staining (inset). Initially RsbS (yellow points) is the only protein getting phosphorylated, while RsbR (blue points) phosphorylation remains low. After about 2 minutes, a sharp increase in the levels of RsbR phosphorylation is detected. The points represent individual measurements of densities from gels, the bars inside the boxes are the mean value and a ± standard deviation of 3 replicates is shown by the error bars, for each time point n=3. The full image of the gel can be found in Supplementary Figure 14. (b) size exclusion chromatography of RsbT. Sample was purified using a GST affinity column and

treated with Prescisison protease (left) after purification on a Superdex S75 column, the tag and protein were successfully separated (right side of the gel) M-marker. (c) RsbRS and RsbT were mixed, and a SEC run was performed to isolate the complex using a Superose 6 Increase column. Analysis of the peak by SDS-PAGE (inset) confirms the presence of RsbT although in low quantities. (d) 2D classes of a negative stain dataset of RsbRST. The red circles on the zoomed in classes indicate putative RsbT density on the periphery of the core closer to the core than the turrets. The scale bar is 200 Å. Uncropped gels can be found in Supplementary figure 14.

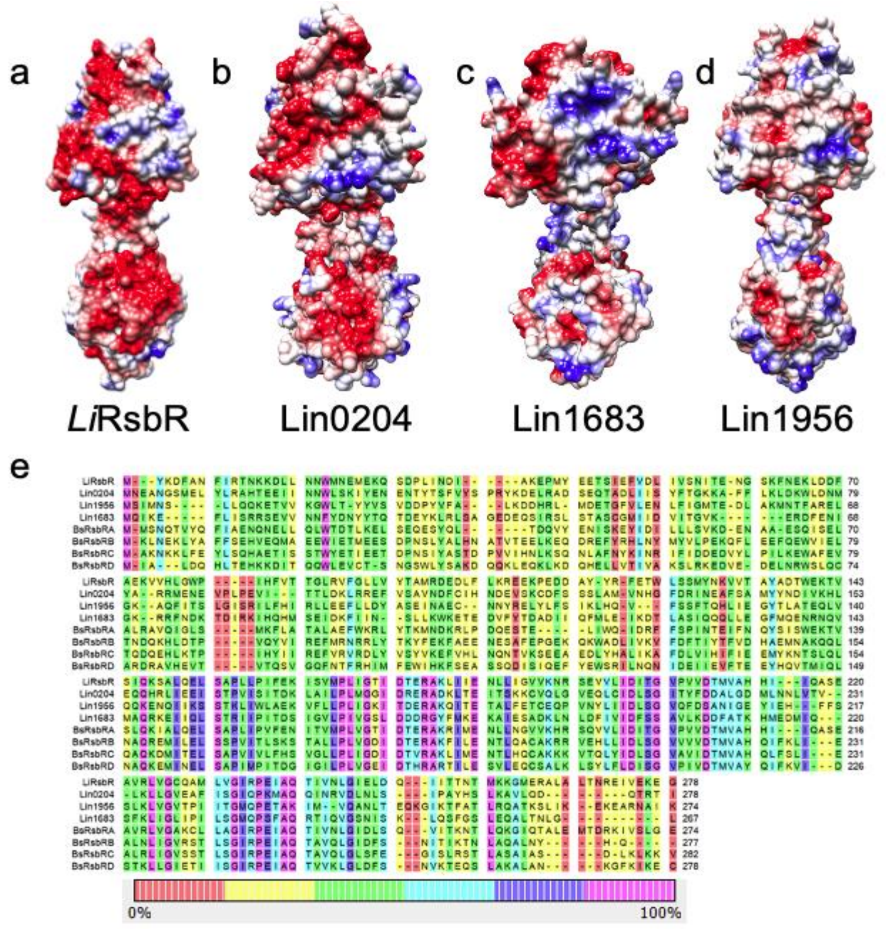

Supplementary figure 12. **Conservation of surface charge properties of the different RsbR paralogs found in *L. innocua*.** Coulombic surface representation of RsbR (a) shows pronounced negatively charged surface (red) on the STAS domain and in parts of the turret close to the STAS domain, indicating a potential strong interaction with RsbT. The other paralogs found in *L. innocua*, Lin0204(b), Lin1683 (c) and Lin1956 (d) have far less pronounced negatively charged patches on the surface of the STAS domain proposed to bind RsbT. (e) Aligned sequences of RsbR paralogs found in the genomes of *L. innocua* and *B. subtilis.* The relative conservation of amino acid residues is depicted by the colors of the heatmap. Conserved amino acids cluster in the C-terminal STAS domain, whereas the amino acids of the N-terminal domains are less conserved (red, yellow and green). Despite

sequence identities between 49% (Lin0204) and 28% (Lin1683), and 25% (Lin1956) the degree of negatively charged surface varies significantly.

a

b

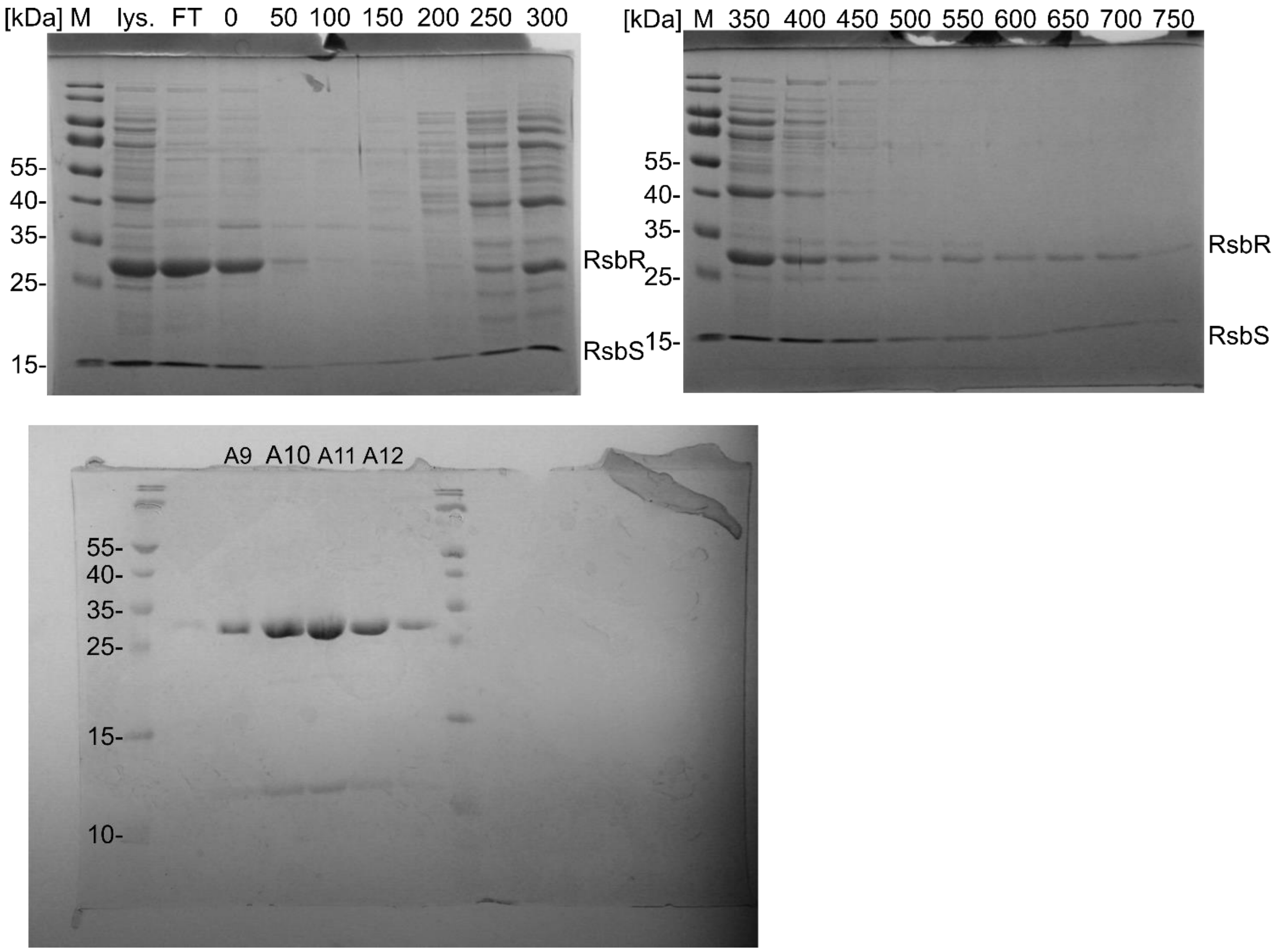

Supplementary Figure 13. **Full size gels from Supplementray figure 2**. (a) Stressosome purification using a DEAE ion exchange column lys. – cell lysate. FT – column flowthrough. The numbers above the lanes indicate the concentration of NaCl (in mM) used to elute the column. (b) Fractions of the RsbRS complex collected from Size exclusion chromatography, the letters indicate fraction names.

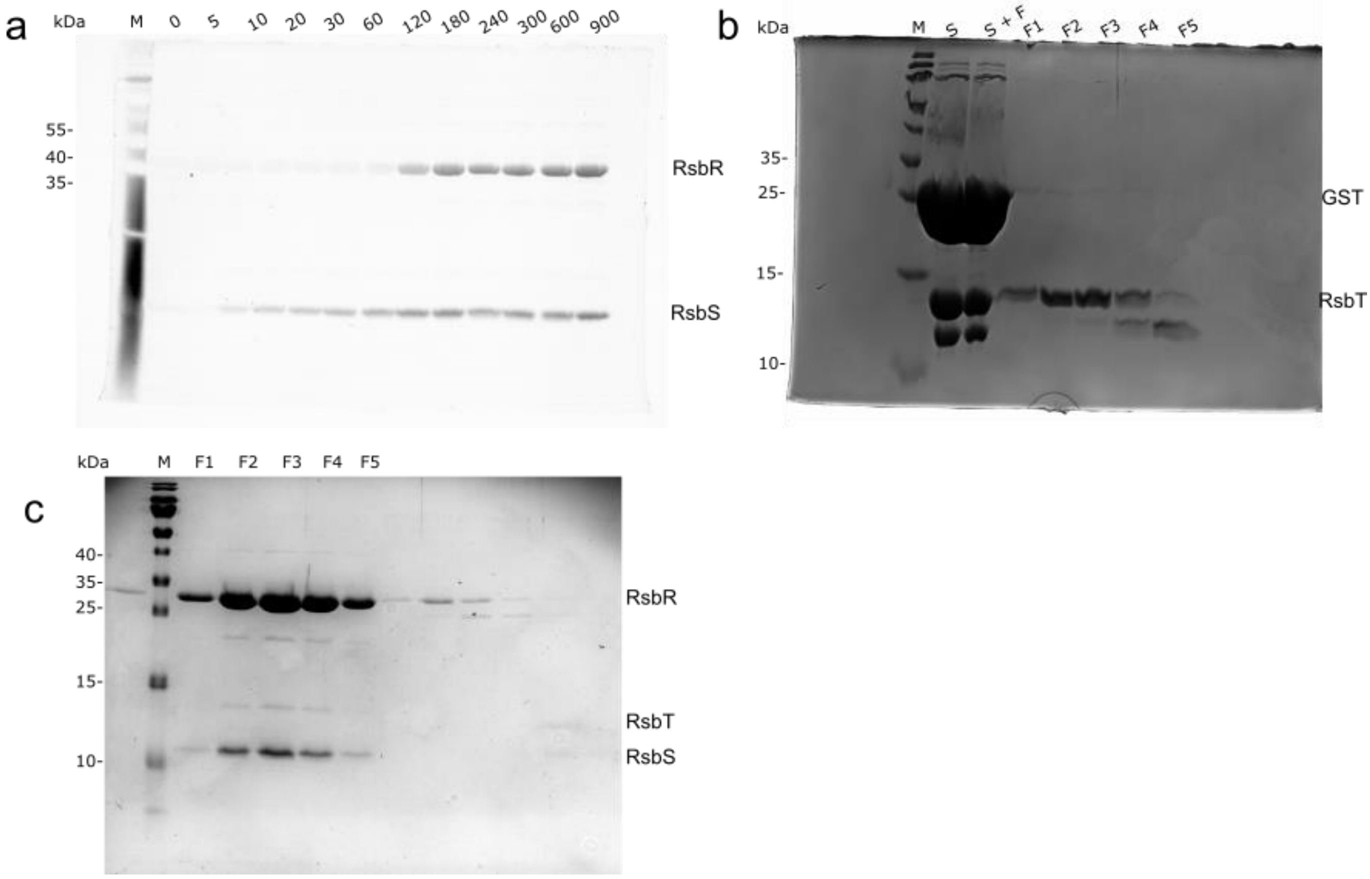

Supplementary Figure 14. **Uncropped gels from Supplementary figure 11.** (a) A phosphorylation assay gel stained in ProQ diamond. The numbers above the lanes indicate time since start of reaction. The numbers on the side show the marker bands. The gel contrast was inverted for easier readability. (b) Gel of a purification of RsbT. S and S+F indicate sample prior and after filtering, respectively. F1-5 are the fractions from SEC chromatography after the protein has been treated with protease. (c) Assembly of a ternary complex of RsbR, RsbS and RsbT. The gel shows fractions from a SEC run (1-5) with the presence of the RsbT band indicated.

Supplementary Table 1: Plasmids and mutant strains lineage used in this study

Plasmid or strain	Reference, source (or lineage[a])
**Plasmids**	
pMAD	(Arnaud, Chastanet, & Débarbouillé, 2004)[1]
pEX-K168::*rsbR1* (T209A)	Eurofins Genomics
pEX-K168::*rsbR1* (T241A)	Eurofins Genomics
pMAD::*rsbR1* (T175A)	(Dessaux et al., 2020)[2]
pMAD::*rsbR1* (T209A)	This study
pMAD::*rsbR1* (T241A)	This study
pET11a-LiRS	(Kaltwasser, 2015)[3]
**Strains**	
Escherichia coli DH-5α	Lab stock
Escherichia coli BL21 Star ™ (DE3)	Thermo Scientific
L. monocytogenes EGD-e WT	K. Boor
L. monocytogenes EGD-e Δ*sigB*	(Guerreiro, Wu, et al., 2020)[4]
L. monocytogenes EGD-e (*rsbR1* (T175A))	(Dessaux et al., 2020)[2]
L. monocytogenes EGD-e (*rsbR1* (T209A))	This study
L. monocytogenes EGD-e (*rsbR1* (T241A))	This study

Supplementary Table 2: Primers used in this study

Primer sequence (5'-3')	Target
AAGAAAAACCTGAAGATGATGC	*rsbR1* (T209A)_F
TACCATAGCGTCTACAACAG	*rsbR1* (T209A)_R
TGGGAAAAGACAGTTTCTATCCA	*rsbR1* (T241A)_F
GTTTACAATAGCTTGAGCG	*rsbR1* (T241A)_R
CACCATGTATAAAGATTTTGCAAACTTCATCCG	*rsbR1*_up_flank_F
**RT-qPCR**	
TGGGGAGCAAACAGGATTAG	16S_F
TAAGGTTCTTCGCGTTGCTT	16S_R
CATATTCGAAGTGCCATTGC	*lmo2230*_F
CTGAACTAGGTGAATAAGACAAAC	*lmo2230*_R
AAAGAACCAAAGGCACCAAC	*inlA*_F
ATTCCCGCCGTTATTTGTTG	*inlA*_R

Supplementary references

1. Arnaud, M., Chastanet, A. & Débarbouillé, M. New vector for efficient allelic replacement in naturally nontransformable, low-GC-content, gram-positive bacteria. *Appl. Environ. Microbiol.* **70**, 6887–6891 (2004).

2. Dessaux, C., Guerreiro, D. N., Pucciarelli, M. G., O'Byrne, C. P. & García-del Portillo, F. Impact of osmotic stress on the phosphorylation and subcellular location of Listeria monocytogenes stressosome proteins. *Sci. Rep.* **10**, 1–15 (2020).

3. Kaltwasser, S. Structural characterization of stressosome complexes by single-particle cryo-electron microscopy. (2016).

4. Guerreiro, D. N. *et al.* Mild stress conditions during laboratory culture promote the proliferation of mutations that negatively affect sigma B activity in listeria monocytogenes. *J. Bacteriol.* **202**, 1–20 (2020).
