# Supplementary material for: Molecular insights into intra-complex signal transmission during stressosome activation
Source: Commun Biol. 2022 Jun 27;5:621. doi: 10.1038/s42003-022-03549-9 (PMC9237128; doi:10.1038/s42003-022-03549-9)
Supplement: Supplementary file 3 — Description of Additional Supplementary Files [file 42003_2022_3549_MOESM3_ESM.pdf]

Description of Additional Supplementary Files

File name: Supplementary Data 1
Description: The raw data of measured gene expression levels of two sigB activity reporter genes in various mutants described in figure 3, panels c and d.

File name: Supplementary Movie
Description: Unsymmetrized map obtained from extensive 3D classification in RELION 3.0, followed by refinement in CryoSparcV2.

File name: Supplementary Movie 2
Description: Variability of Page 6 of 19 conformations in the RsbR helical linker region within the dataset of LiRsbRS as obtained by 3D variability analysis. The movie is composed of changes between 40 reconstructed cryoEM maps.

File name: Supplementary Movie 3
Description: A morph between the different conformations seen in LiRsbR and LiRsbS STAS domains. The RsbR STAS domain is colored blue, RsbS is colored yellow.
